# The role of the cytochrome P450 superfamily in the skin

**DOI:** 10.1017/erm.2024.5

**Published:** 2024-04-15

**Authors:** Qianqian Chen, Tuan Wang, Xia Wu, Huipu Yuan, Yuan Wei, Ying Xiao

**Affiliations:** 1 School of Pharmacy, Jiangsu University, Zhenjiang, China; 2 The Fourth School of Clinical Medicine, Zhejiang Chinese Medical University, Hangzhou, China; 3 Affiliated Hangzhou First People's Hospital, School of Medicine, Zhejiang University, Hangzhou, China; 4 Dermatology Department, Sir Run Run Shaw Hospital, Hangzhou, China; 5 Sir Run Run Shaw Hospital, School of Medicine, Zhejiang University, Hangzhou, China

**Keywords:** CYP450, drug development, skin biology, skin disease, toxicology

## Abstract

In mammals, the skin acts as a barrier to prevent harmful environmental stimuli from entering the circulation. CYP450s are involved in drug biotransformation, exogenous and endogenous substrate metabolism, and maintaining the normal physiological function of the skin, as well as facilitating homeostasis of the internal environment. The expression pattern of CYP450s in the skin is tissue-specific and thus differs from the liver and other organs. The development of skin topical medications, and knowledge of the toxicity and side effects of these medications require a detailed understanding of the expression and function of skin-specific CYP450s. Thus, we summarized the expression of CYP450s in the skin, their function in endogenous metabolic physiology, aberrant CYP450 expression in skin diseases and the influence of environmental variables and medications. This information will serve as a crucial foundation for future studies on the skin, as well as for the design and development of new drugs for skin diseases including topical medications.

## Introduction

The skin consists of the epidermis, dermis and subcutaneous layer, and it is the most important barrier between the external environment and the internal environment of the organism. In addition to being one of the primary organs involved in extrahepatic drug metabolism, each layer of the skin has a specific function, helping to shield the organism from external stimuli. Cytochrome P450 enzymes, otherwise named CYP450s or cytochrome P450s, act as a metabolic barrier in the skin. The CYP450 component of the metabolic barrier dynamically controls its homeostasis by activating or inactivating physiologically important molecules (Ref. 1). The broad superfamily of CYP450 monooxygenases is composed of self-oxidizing haeme proteins. Their distinctive 450 nm absorption peak gave rise to their name. CYP450s include the CYP1, CYP2 and CYP3, etc., a large number of enzyme families. The majority of drugs are metabolized by CYP450s because these enzymes can convert drugs from hydrophobic to hydrophilic forms, which are more easily excreted from the body. Therefore, CYP450s are responsible for the metabolism of a wide range of endogenous and exogenous substances, including fatty acids, prostaglandins, vitamin A, vitamin D, and steroids.

Herein, upon a thorough literature review, this paper summarized the expression of CYP450s in the skin, their function in endogenous and exogenous metabolic physiology, aberrant CYP450 expression in skin diseases, and the influence of environmental variables and medications.

CYP450s are a major source of variation in drug pharmacokinetics and drug response. A significant issue in clinical practice is individual variations in the responses to drugs, which may result in drug toxicity or lead to poor drug efficacy. Understanding drug response variability, improving therapeutic efficacy and lowering the occurrence of drug toxicity can all be achieved through the study of pharmacokinetic processes (Ref. 2).

Phases I and II are the two primary divisions of drug metabolism. Phase I metabolic reactions are the rate-limiting steps in drug elimination from the organism, and these reactions can cause detoxification or toxicity-enhancing effects. Of the 57 functional CYP450s in humans, approximately 12 enzymes are members of the CYP1, CYP2 and CYP3 families, which biotransform the majority of foreign substances. The activity of these three families accounts for 70%–80% of metabolic activity, mainly through direct effects on hydroxylation, epoxidation, dealkylation, oxidation and dehalogenation (Ref. 3). Drugs and their metabolites are then mixed with endogenous molecules for subsequent elimination from the body through phase II metabolic processes, which have a detoxifying effect. However, some of the active metabolites that are generated may harm the liver (Ref. 4). As CYP450s are the primary cause of drug pharmacokinetic variability and drug response variability, CYP450 research is important for clinical medication recommendations.

## CYP450 expression in healthy skin tissue and the role of CYP450s in skin metabolism

### Cyp450 expression in healthy skin tissue

The expression of CYP450s varies depending on the tissue and cell type. For example, the P450 isoform CYP3A4 is most abundantly expressed in the human liver, whereas it is only very weakly expressed in the human skin (Ref. 5). The expression of CYP450s is affected by a variety of factors, which include induction by exogenous substances, hormonal regulation, genetic polymorphisms, cytokines, disease state, sex, and age, amongst others. The skin is the outermost layer of the human body, and it allows for direct communication with the environment and external substances, as well as the absorption and metabolism of exogenous substances. Understanding the precise expression and function of CYP450s in the skin is a crucial prerequisite for the study of skin metabolism because it is still unclear how these crucial metabolic enzymes function in the uptake, metabolism and elimination of exogenous substances and endogenous substrates in the skin.

**Figure 1.**
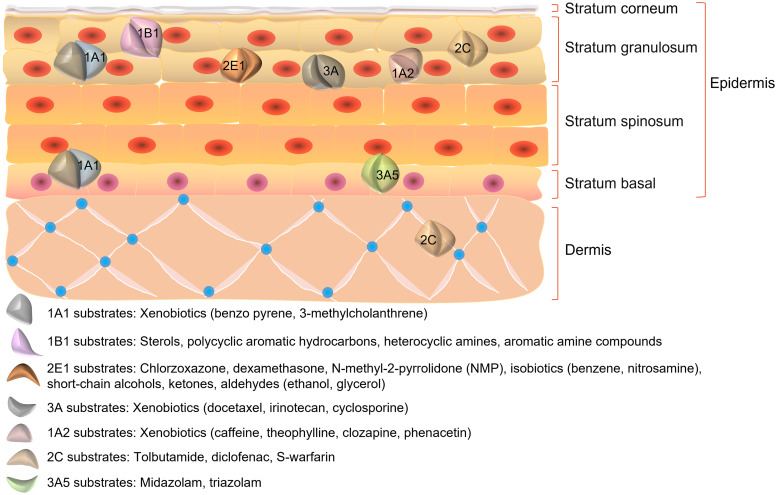
Structure of the skin and distribution of CYP450s.

Different CYP450 isoforms have distinct expression patterns and play different roles in the skin. The human skin most abundantly expresses CYP1B1, followed by CYP2E1 at a significantly higher level than CYP1A1. CYP1A2, an enzyme that is closely related to CYP1A1, is expressed at a similarly low level. According to immunocytochemical investigations, CYP1A1, CYP2B6, CYP2E1 and CYP1B1 are predominantly expressed in keratin-forming cells (Ref. 6), while CYP3A5 expression is primarily confined to the basal layer. CYP3A4 is also strongly expressed in endothelial cells of the inner vascular layer within the connective tissue of the human skin. Additionally, it was discovered that a new dioxin-induced CYP450, CYP2S1, is expressed in the human skin. These findings suggest that CYP450s are variably expressed in the skin and may have different functions (Ref. 7). The distribution of CYP450s and the fundamental structure of the skin are shown in Figure 1.

### Role of CYP450s in skin metabolism

As the largest organ in the human body, the skin participates in the general metabolic process of the human body. The basal layer of cells absorbs nutrients and oxygen, divides into daughter cells and pushes upward until the stratum corneum dies. The results of a previous study demonstrated that a variety of CYP450-dependent metabolic enzymes are expressed by the cells that form keratinocytes in the human skin, and that these enzymes are capable of active uptake, biotransformation and anti-transport of a variety of exogenous substances, such as drugs, solvents and carcinogens (Ref. 8). Several studies have revealed that xeno-metabolizing enzymes and transport proteins can function as a second skin barrier in addition to the stratum corneum, which is an important structural layer in the epidermis.

Fatty acids, steroids, prostaglandins and other natural and manufactured bioactive compounds are catabolized by CYP450s (Ref. 9). Many different CYP450s are expressed by human skin cells, both in vivo and in three-dimensional organ preparations. The basal epidermal keratinocytes, small sweat glands and sebaceous glands express CYP26AI, which is responsible for the metabolism of retinoic acid in skin cells. Moreover, CYP4F22, an ultra-long-chain fatty acid hydroxylase that produces acyl ceramide, is crucial for the development of skin permeability (Ref. 10).

Furthermore, CYP450s are crucial for the metabolism of several vitamins. Retinoids are 3,4-desaturated by CYP27C1 in the skin, which is crucial for preserving the level of active retinol in the skin (Ref. 11). In vitro synthesis of vitamin A1 into vitamin A2 in keratinocytes has been demonstrated to be significantly mediated by CYP27C1 (Ref. 12). In addition, it has been discovered that CYP11A1, a protein found in the human serum that plays a catalytic function in the first step of steroidogenesis, also impacts vitamin D3 and vitamin D2. Moreover, CYP24A1, CYP27A1, CYP27B1, CYP3A4 and CYP11A1 are crucial for the metabolism of vitamin D in the blood (Ref. 13).

Taken together, the evidence suggests that CYP450s play a key role in skin metabolism. They are involved in the catabolism of biologically active substances, such as fatty acids, steroids and retinol, amongst others, and also in the metabolism of various vitamins, such as vitamin A and vitamin D. Table 1 shows the function and substrates, as well as the location of major CYP450s expressed in the skin.

**Table 1.** Major CYP450s in the skin

CYP	Function	Substrates	References	Cell types
1A1	Catalyzes the bioactivation of polycyclic aromatic hydrocarbons (PAH) and initiates mutagenesis and carcinogenesis	Xenobiotics (benzo pyrene, 3-methylcholanthrene)	CYP1A1 plays a role in normal skin barrier function, and its enzymatic activity is a critical regulator of beneficial aryl hydrocarbon receptor (AHR) signalling in the context of skin inflammation (e.g., psoriasis) (Refs 14, 15, 16)	Keratin-forming cells
1A2	Participates in precarcinogen activation (mycotoxins, aflatoxins, nitrosamines, etc.) and drug metabolism, and responsible for the hydroxylation process of endogenous hormones	Xenobiotics (caffeine, theophylline, clozapine, phenacetin)	CYP1A2 activity in the blood is strongly induced after acute dioxin exposure (Refs 17, 18), and its absence reduces skin Benzo[a]pyrene (BAP) adduct levels (Ref. 19)	keratin-forming cells
1B1	Catalyzes epoxidation and hydroxylation of procarcinogens, and interacts with many nuclear receptors such as peroxisome proliferators-activated receptors (PPARs)	Sterols, polycyclic aromatic hydrocarbons, heterocyclic amines, aromatic amine compounds	CYP1B1 plays a significant role in bioactivation and carcinogenesis of dibenzo[def,p]chrysene (DBC) in a mouse skin tumour model (Ref. 20), and mediates the first epoxygenation (Refs 21, 22)	keratin-forming cells
2E1	Participates in catalyze the metabolism of various endogenous and exogenous molecule compounds and plays a major role in the biological activation of many carcinogens and detoxication (Ref. 23)	Chlorzoxazone, dexamethasone, N-methyl-2-pyrrolidone (NMP), isobiotics (benzene, nitrosamine), short-chain alcohols, ketones, aldehydes (ethanol, glycerol) (Ref. 24)	CYP2E1 is activated in proinflammatory environments or infectious conditions (Ref. 23), is significantly reduced in melanoma tissues, is involved in the metabolism of dexamethasone (Refs 25, 26), and to a lesser extent in humans (Ref. 27)	Keratin-forming cells
2A6	Catalyzes cholesterol, steroids, and other lipid synthesis reactions and the oxidation of nicotine and the activation of carcinogens (Ref. 28); metabolizes ethanol and procarcinogens (e.g., nicotine, nitrosamines) and drugs	Nicotine, arachidonic acid, progesterone, aflatoxin B1, nitrosamine, benzophenone, coumarin, etc. (Ref. 29)	CYP2A6 is possibly involved in the pathogenesis of drug-induced allergic skin reactions (Ref. 30)	Keratin-forming cells
3A	Catalyzes three steroid hormones by 6β-hydroxylation (Ref. 31) and participates in drug metabolism and biotransformation	Xenobiotics (docetaxel, irinotecan, cyclosporine)	CYP3A participates in the metabolic activity of human skin and tissue-engineered skin equivalents (Ref. 6)	Keratin-forming cells and endothelial cells
11A1	Catalyzes cholesterol conversion to pregnenolone which is the first step in steroidogenesis, (Ref. 13) and metabolizes vitamin D3 (Ref. 32)	Cholesterol, 7-dehydrocholesterol, lumefantrine, hydroxysterols, etc.	CYP11A1 plays critical roles in the skin through its initiation of steroidogenesis and metabolism of vitamin D, etc. (Ref. 33), and it may play an anti-photocarcinogenic role (Refs 32, 34, 35)	Epidermal keratinocytes and melanoma cells
2J	Metabolize AA into epoxyeicosatrienoic acids (EETs) and hydroxyeicosatetraenoic acids (HETEs)	Arachidonic acid (AA), linoleic acid (LA), and other lipids and xenobiotics	Play a role in fatty acid metabolism to produce biologically active compounds, with CYP2J8 being most highly expressed in the skin in mice (Ref. 36)	No report

## Role Of CYP450s in the response to environmental stimuli

### Changes in CYP450 expression in response to toxic compounds

Despite the stratum corneum serving as an epidermal barrier and the skin serving as a physical and biochemical barrier that effectively protects the organism from the environment, potentially harmful environmental substances are still absorbed by the organism. It has been demonstrated that cells exposed to chlorpyrifos dramatically influence the expression of all investigated CYP450 isoforms. A previous study showed that CYP27A1 mRNA levels were decreased in chlorpyrifos-treated HaCaT keratinocytes, although CYP27B1 and CYP24A1 mRNA levels were elevated. Further research is required to determine how chlorpyrifos works because it may affect how vitamin D3 is metabolized in skin cells (Ref. 33).

Exogenous metabolic enzymes and transport proteins act as a second biochemical barrier to the skin. The interaction between small molecule compounds and target molecules in separation, after penetration, participates in the reaction by oxidation, chemical activation or inactivation of exogenous compounds. CYP450s are the most important enzymes involved in this process. In addition to their detoxification function, CYP450s may be involved in allergic reactions to low-molecular-weight substances, such as in contact dermatitis.

### Protective effects of skin CYP450s against damage from ultraviolet radiation and oxidative stress

Sunlight is essential for human survival; however, ultraviolet radiation can have a negative impact on human health. Cytotoxic reactive oxygen species (ROS) are created by solar energy and ultraviolet light. The expression of antioxidant enzymes in the skin, such as superoxide dismutase and catalase, is induced as an adaptive response to repetitive ultraviolet exposure to neutralize ROS and safeguard cells from oxidative stress. The findings imply that solar ultraviolet radiation exposure of the human skin or cultured keratinocytes increases CYP1A1 and CYP1B1 mRNA and protein expression, and may increase the biological activity of PAHs or other environmental contaminants. Pretreatment of keratinocytes or cell lines (HaCaT cells) with the antioxidant compound N-acetylcysteine or the potent AHR antagonist naphthoflavone significantly inhibited the induction of CYP1B1 mRNA by ultraviolet B, and these data suggest that ultraviolet B photoproducts and AhR are involved in CYP1B1 induction (Ref. 37).

CYP1A1 expression in healthy skin is extremely low, while CYP1B1 expression is more prevalent. Topical application of coal tar induces CYP1A1 expression in the human skin, and it has also been discovered that CYP2E1 expression is significantly reduced in the skin by coal tar. This is because CYP2E1 is involved in the defense against ROS in healthy skin, whereas this defense is diminished in people with psoriasis. It is also known that the peri-lesional or non-lesional skin of people with psoriasis is more prone to irritation following coal tar or dibenzylphenol treatment than lesional skin, as well as being more vulnerable to phototherapy-induced erythema (Ref. 5).

### Effect of medications on skin CYP450 expression

A previous study showed that when administered systemically, dexamethasone, a widely used topical drug in dermatology, induces the hepatic production of multiple CYP450 isoenzymes (Ref. 26). The study also showed that after transdermal administration of dexamethasone, the expression of CYP1A1, CYP2B6, CYP2E1 and CYP3A, as well as multidrug resistance-associated protein (MRP)1, MRP13 and MRP15, and multidrug resistance 1 (MDR1) protein, was upregulated. Immunohistology showed that CYP1A1, CYP2B6, CYP2E1 and CYP3A, as well as MRP1 and MDR1, demonstrated positive immunofluorescence in skin specimens. The constitutive activity of CYP1A1, CYP2B, CYP2E1 and CYP3A was also demonstrated using human skin keratinocytes, and their expression increased by 2–10 fold. These findings imply that a range of transport-associated and detoxification-metabolizing CYP450s are expressed by human skin keratinocytes (Ref. 6).

We can conclude that skin CYP450s play a key role in response to environmental stimuli, and participate in the metabolism of compounds through oxidative reactions and chemical activation. Moreover, they can protect the skin from damage by ultraviolet radiation and oxidative stress.

## Abnormal expression and role of CYP450s in skin diseases

### The expression of CYP450s in psoriasis

Inflammatory cytokines, including interleukin (IL)-8, IL-6, IL-1, IL-18, IL-12, tumour necrosis factor-*α* and interferon-*γ*, are found in excess in people with psoriasis, which is a chronic inflammatory illness that primarily affects the skin and joints (Ref. 38). Oxidative stress may significantly influence the pathogenesis of psoriasis. Glutathione S-transferase constitutes a group of antioxidant enzymes, and CYP450s can influence oxidative and reductive reactions. In view of this evidence, the potential role of CYP450s in the pathogenesis of psoriasis can be investigated. Clinically, treatments for patients with mild psoriasis include topical corticosteroids, vitamin D analogues, calmodulin neural phosphatase inhibitors, keratolytic medicines and targeted phototherapy (Ref. 39).

Furthermore, genes controlling aberrant keratinocyte differentiation and epidermal barrier function (*CYP4F22*, *SULT2B1*) are upregulated in psoriasis and may represent drug targets in psoriatic skin. These genes also show opposing expression patterns in psoriasis (Ref. 40). Downregulation of several CYP450s is associated with increased cytokine levels in inflammatory disorders; for instance, following therapy with some cytokines, CYP450 enzyme levels may be restored, resulting in protein-mediated drug interactions (Ref. 41).

Researchers have found that CYP1A1 and CYP2E1 are more highly expressed in the skin of people with psoriasis, both before and after phototherapy, indicating that psoriatic lesions respond differently to oxidant exposure. Nevertheless, CYP1B1 expression was higher in treated psoriatic tissues than in control tissues as a result of increased free radical production by activated neutrophils or exposure to ultraviolet light to preserve the antioxidant capability in psoriasis. These findings imply that, despite the fact that phototherapy has no effect on these enzyme systems, psoriasis is an oxidative stress-related disease that would benefit from further investigation (Ref. 42).

### Effect of ultraviolet irradiation on skin CYP450s

Immunosuppression and ultraviolet-induced DNA damage are both known to enhance the risk of skin tumours. CYP11A1 in the skin, which is involved in vitamin D metabolism, creates 20-hydroxyvitamin D3 (referred to as 20OHD). Researchers have experimentally demonstrated that the use of 20OHD dramatically reduces skin oedema and wrinkles caused by ultraviolet exposure, suggesting that CYP11A1 inhibits aging and wrinkles caused by ultraviolet exposure and has a photoprotective effect. Other studies have shown that the specificity of vitamin D, lumefestrol and 7-dehydrocholesterol, as well as the beginning of topical steroid production, makes CYP11A1 an efficient enzyme that protects against damage caused by ultraviolet radiation, and that disruption of its activity results in skin pathology, indicating that the skin plays a significant role (Ref. 33).

Another element controlling CYP450 expression in the skin is ultraviolet radiation. Exposure of human skin to ultraviolet radiation results in the elevation of CYP1A1 and CYP1B1 mRNA and protein expression, with ultraviolet B dose- and time-dependent induction (Ref. 43). After topical application of crude coal tar and exposure to medium-wave ultraviolet B, the activity of aryl hydrocarbon hydroxylase, 7-ethoxyresorufin-O-deethylase and 7-ethoxycoumarin-O-deethylase in the skin of neonatal rats increased in a dose-dependent manner. Treatment of animals with coal tar followed by UVB irradiation leads to increased CYP450-dependent activity in the skin (Ref. 44). As mentioned previously, in non-ultraviolet-irradiated skin, CYP1A1 is mainly localized in the basal cell layer of the epidermis, whereas CYP1B1 is mainly localized in the epidermal cells rather than in the basal cell layer, suggesting that ultraviolet B-mediated induction of CYP1A1 and CYP1B1 in human skin may lead to enhanced bioactivity of some environmental pollutants, thus making the human skin more susceptible to ultraviolet B-induced skin cancer or allergic and irritant contact dermatitis (Ref. 45).

### Abnormal expression of CYP450s in other skin diseases

Bowen-like papulosis (BP) is a benign and potentially carcinogenic disease associated with human papillomavirus infection, but the underlying mechanisms remain unclear. The metabolism of exogenous substances by CYP450s is greatly dysregulated in patients with BP compared with healthy controls according to a recent study comparing skin samples from healthy subjects with those from patients with BP. Therefore, targeting these signals may be a potential approach to BP treatment.

Damage to the skin barrier can lead to the development of a variety of dermatological conditions, such as ichthyosis and atopic dermatitis. Acyl ceramides are an important component of the skin barrier; however, the mechanism of their production has not yet been clarified. CYP4F22 is a type I membrane protein found in the endoplasmic reticulum. It functions as an ultra-long-chain fatty acid omega-hydroxylase that catalyzes the production of acyl ceramides, which are vital in the construction of the skin barrier. *CYP4F22* is one of the autosomal recessive genes for congenital ichthyosis, and lipid studies in patients with ichthyosis have revealed that acyl ceramide formation in the skin is substantially decreased in these individuals (Ref. 10). Therefore, it appears that epidermal damage in people with ichthyosis is related to CYP4F22 deficiency.

Kava dermopathy is a typical cutaneous reaction to kava, a popular Pacific beverage. Kava dermopathy exhibits clinical similarities to autosomal recessive congenital ichthyosis, predominantly platysmal ichthyosis. Similar to the genetic problems in lamellar ichthyosis type 3, the pathophysiology of kava disease may be linked to deficiencies in the function of one or more CYP450s associated with epidermal integrity (Ref. 46).

The skin, like the liver, plays an essential role in ethanol and acetylcholinesterase metabolism, and if its metabolic pathways are disrupted, there is an increased risk of developing ethanol/acetylcholinesterase-related dermatoses. Malignant melanoma is a highly lethal form of skin cancer that is becoming more common worldwide. Data have shown that three ethanol-metabolizing enzymes (including P4502E1 and catalase, etc.) and one acetylcholinesterase-metabolizing enzyme (aldehyde dehydrogenase 2) are clearly downregulated in melanoma tissues (Ref. 11).

Given this evidence, it is easy to comprehend how different skin conditions affect the expression of CYP450s. For instance, psoriasis is characterized by changes in the expression of CYP450s associated with inflammatory factors, lipid metabolism, and oxidative stress. With exposure to ultraviolet radiation, CYP450s can partially protect the skin, whereas some skin conditions are characterized by more significant changes in CYP450 expression. This knowledge might be useful for the development of skin medications.

## Discussion

As An important metabolizing enzyme, CYP450 plays an essential role in drug transformation, absorption, elimination and detoxification (Ref. 47). Thus, CYP450s are a major source of variation in drug pharmacokinetics and drug response. A significant issue in clinical practice is individual variations in the responses to drugs, which may result in drug toxicity or lead to poor drug efficacy. For example, hepatic CYP1A2 mRNA and protein expression varies between 15- and 40-fold between individuals (Ref. 48), and CYP1A2 activity is significantly higher in men than in women. The antipsychotic drug clozapine was mainly metabolized by CYP1A2. The steady-state plasma concentrations of clozapine showed up to 45-fold variability, which was higher in women (Ref. 49). Therefore, the difference in the expression of CYP1A2 enzyme caused by gender difference leads to variations in the metabolism of drugs such as clozapine, resulting in different drug efficacy.

The most prevalent and important drug-metabolizing enzymes in the mammalian skin, which are activated in response to xenobiotic exposure, are the CYP450 superfamily. CYP450s play numerous important roles in the metabolism of both endogenous and exogenous substrates in the skin, including vitamin A and vitamin D, fatty acids, sterols and medicines. In addition, the expression of numerous CYP450s is markedly increased in some skin conditions, such as psoriasis. CYP450 enzymes are targets of particular interest when developing drugs for use in skin diseases, because most of the available drugs in dermatology are either substrates, inducers, or inhibitors of this family of enzymes. This review integrates the existing knowledge about CYP450s in the skin. However, further studies are needed to establish the functional significance of drug metabolism in the skin, as well as the function of CYP450s in dermatopathology and treatment. The development of novel and more effective treatments for the increasing number of skin illnesses will be made possible by understanding the bioavailability of skin medications, especially the mechanism of action of CYP450s in the skin.

CYP450s are widely found in the skin and play important roles in the metabolism of both endogenous and exogenous substrates in the skin, including medicines. The expression of P450 in the skin changed in response to external stimuli or environmental changes to protect the skin. For example，as mentioned above, CYP11A1 was an efficient enzyme that played critical roles in the skin through its initiation of steroidogenesis and metabolism of vitamin D, etc., thus, protecting skin against damage caused by ultraviolet radiation. Therefore, the treatment of skin aging and damage caused by photoaging can be considered to improve the activity of CYP11A1 as a target. Besides, there is corresponding finding in clinical trial, using CYP450 inhibitor Ketokonazol to investigate the antipsoriatic effects combined with UVB-radiation treatment (NCT00678756). In addition, the expression of CYPs was changed in disease states such as psoriasis. The expression trend of different CYP isoforms was different before and after treatment in psoriasis patients, some clinical trials show that there is a change of CYP450s as a consequence of drug interaction including psoriasis (NCT02993471, NCT02397382 and NCT03718884), suggesting that P450s may be therapeutic targets and provide new ideas for the treatment of skin diseases. Meanwhile, the changes of CYP450s should also be paid attention to in the existing treatments. Combined with CYP450s-related drugs and existing therapeutic measures applied to clinical diagnosis and treatment will provide a new idea for the effective treatment of skin diseases.
